# Proteomic identification of host and parasite biomarkers in saliva from patients with uncomplicated *Plasmodium falciparum* malaria

**DOI:** 10.1186/1475-2875-11-178

**Published:** 2012-05-28

**Authors:** Honglei Huang, Mukram M Mackeen, Matthew Cook, Eniyou Oriero, Emily Locke, Marie L Thézénas, Benedikt M Kessler, Davis Nwakanma, Climent Casals-Pascual

**Affiliations:** 1 Wellcome Trust Centre for Human Genetics and Centre for Cellular and Molecular Physiology, Roosevelt Drive, Oxford, OX3 7BN, UK; 2 MRC Laboratories, Banjul, The Gambia; 3 The PATH Malaria Vaccine Initiative, Washington, DC, 20001, USA

## Abstract

**Background:**

Malaria cases attributed to *Plasmodium falciparum* account for approximately 600,000 deaths yearly, mainly in African children. The gold standard method to diagnose malaria requires the visualization of the parasite in blood. The role of non-invasive diagnostic methods to diagnose malaria remains unclear.

**Methods:**

A protocol was optimized to deplete highly abundant proteins from saliva to improve the dynamic range of the proteins identified and assess their suitability as candidate biomarkers of malaria infection. A starch-based amylase depletion strategy was used in combination with four different lectins to deplete glycoproteins (Concanavalin A and *Aleuria aurantia* for *N*-linked glycoproteins; jacalin and peanut agglutinin for *O*-linked glycoproteins). A proteomic analysis of depleted saliva samples was performed in 17 children with fever and a positive–malaria slide and compared with that of 17 malaria-negative children with fever.

**Results:**

The proteomic signature of malaria-positive patients revealed a strong up-regulation of erythrocyte-derived and inflammatory proteins. Three *P. falciparum* proteins, PFL0480w, PF08_0054 and PFI0875w, were identified in malaria patients and not in controls. *Aleuria aurantia* and jacalin showed the best results for parasite protein identification.

**Conclusions:**

This study shows that saliva is a suitable clinical specimen for biomarker discovery. Parasite proteins and several potential biomarkers were identified in patients with malaria but not in patients with other causes of fever. The diagnostic performance of these markers should be addressed prospectively.

## Background

Malaria is an important public health problem causing an estimated 216 million cases worldwide and approximately 655,000 deaths yearly. Most of these deaths occur in sub-Saharan Africa and in children under five years of age [1]. The current standard method to diagnose malaria infection requires the visualization of the parasite in blood using light microscopy. In addition to the discomfort caused by blood sampling, the use of invasive methods to diagnose malaria results in decreased study completion rates in community studies when repeated sampling is necessary. Saliva is a readily accessible, non-invasive body fluid increasingly used as a diagnostic tool. A previous study in Gambian children showing *Plasmodium falciparum* DNA in saliva from malaria patients underscores the suitability of this biofluid to diagnose malaria. In this study, saliva could correctly identify up to 82% of microscopy-positive samples [2].

The human saliva protein composition (proteome) provides a suitable alternative to blood components for biomarker discovery [3]. The salivary proteome has been effectively used to identify markers of human disease such as oral cancer and Sjögren’s syndrome [4,5].

Salivary secretions are a highly complex mixture of proteins such as hormones and enzymes, lipids, carbohydrates and ions that contribute to the many roles and functions of saliva. The composition of saliva results from the contribution of the salivary glands, oral tissues and oral micro-organisms [6]. The application of mass spectrometry and biochemical techniques to salivary samples has enabled the quantitative and qualitative analysis of saliva [7]. To date, more than 1,400 proteins have been identified in saliva. These salivary proteins have been categorized into six structurally related groups: histatins, proline-rich proteins (acidic, basic and glycosylated), statherins and cystatins [8].

The complexity and the high dynamic range of salivary proteins are the main obstacles to identifying potential biomarkers in saliva using mass spectrometry as evidenced by highly-abundant proteins such as amylase, which constitutes approximately 60% of the protein composition of saliva [3,5,6]. Thus, the depletion of highly abundant proteins prior to mass spectrometry analysis is critical to maximize detection of less abundant proteins that may be differentially expressed in response to malaria infection.

Lectins are carbohydrate-binding proteins that reversibly bind to specific mono- and oligosaccharides [9,10]. The reversibility of the lectin-sugar interactions makes them appropriate for enrichment/depletion strategies.[9] In this context, the differential binding affinities of lectins makes them an effective tool for glycoproteomic research. For example, concanavalin A (ConA) binds with high mannose type N-glycans, whereas jacalin (Jac) selectively binds immunoglobulins [11,12].

In this study, the potential of biochemical enrichment methods of salivary proteins to identify potential biomarkers of malaria were optimized to enrich for *Plasmodium falciparum* proteins.

## Methods

### Study population

Matched samples of blood and saliva were collected from children aged ≥10 years with suspected malaria infection and referred for blood film microscopy at the outpatient clinic facility of the Medical Research Council in The Gambia. Patients were prospectively enrolled before determination of malaria status. Saliva samples (2 ml) were collected into separate aseptic Sterilin bottles (Barloworld Scientific). All samples were kept at 4°C–8°C.

**Figure 1:**
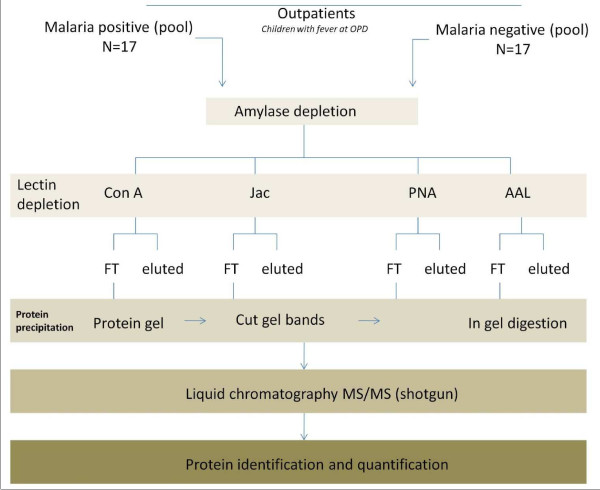
**Study outline.** OPD: outpatient department, ConA: Concanavalin A, Jac: Jacalin, PNA: peanut agglutinin, AAL: *Aleuria aurantia*, FT: flow-through fraction, MS/MS: tandem-mass spectrometry.

Saliva samples from 34 children with suspected malaria were used for proteomic profiling studies. The sample workflow is outlined in Figure 1. Of these samples, 17 were confirmed as malaria positive and 17 as malaria negative by light microscopy and by PCR as detailed elsewhere [2]. All patients gave informed consent, and parental or guardian consent was obtained for participants aged 18 years or younger. The study was jointly reviewed and approved by the Gambian Government–Medical Research Council Laboratories Ethics Committee and the Western Institutional Review Board and the PATH Research Ethics Committee.

### Amylase depletion

Amylase depletion was carried out following the protocol reported by Deutsch *et al.*[3]. Briefly, pooled saliva samples (N = 17) were centrifuged at 12,000 × *g* for 5 min at room temperature (RT) to remove insoluble cell debris and food remnants. A 1 ml syringe (BD, REF 309002) with a 0.45 μm tip filter (Life Science, PN K556T) was filled with 100 mg of potato starch (Sigma S4251) per 100 μl saliva sample. The starch was moisturized with 500 μl of distilled water by hand pressing (20 s) through the device. Saliva samples were loaded in a 1:1 ratio with starch and hand pressed in the filter for 120 s. The flow-through was collected and then the starch was eluted in a column with 1X SDS-PAGE loading buffer. Amylase depletion was confirmed by band visualization in a silver-stained SDS protein gel (Additional file 1). Initially, the ratio of saliva to starch was optimized by testing the depletion of amylase at various volumes of saliva sample. The starch was fixed at 100 mg and used to deplete 50, 100, 200, 300, 400 μl of saliva sample.

The volume of lectin beads was fixed at 50 μl and used to deplete 50, 100, 200, 300, 400 μl of amylase-depleted saliva samples. Each fraction, flow through (FT), wash (W), elute (E) and SDS-elute (SDS) were collected and analysed by SDS-PAGE.

### Glycoprotein depletion using lectins

Glycoprotein enrichment/depletion was performed according to the manufacturer’s instructions for all lectins and additionally for ConA as described by Alonzi *et al.* for ConA [13].

Concanavalin A (ConA) (Sigma Aldrich, Steinheim, Germany) (100 μl): lectin beads were pre-equilibrated with 2 × 1 ml H_2_O (Milli-Q) followed by 1 ml of 1 mM MgCl_2_, 1 mM CaCl_2_ and 1 mM MnCl_2_ in water. The beads were then washed with 2 × 1 ml 50 mM Tris/HCl pH 7.2 prior to the addition of 100 μl of the starch-depleted sample. The sample was incubated at 4°C for 1 h on a rotator. The supernatant was then collected by centrifugation at 1,500 rpm. The pellet was washed with 1 ml 50 mM Tris/HCl buffer pH 7.2 and the supernatant was collected. The pellet was washed again with 1 ml water but the supernatant was not collected. 1 ml of hot (70°C) 0.2 M α-methyl-mannoside was used to elute bound glycoproteins and the supernatant was collected. A second elute with 1 ml of hot (70°C) 0.2 M α- methylglucoside was used and the supernatant was collected before a final elution with 50 μl 2X SDS loading buffer.

*Aleuria aurantia* (AAL) (Vectors laboratories, Burlingame, CA, USA) (100 μl): The lectin beads were pre-equilibrated with 2 × 1 ml 10 mM HEPES pH 7.5 followed by 1 ml of 1 mM MgCl_2_, 1 mM CaCl_2_, and 1 mM MnCl_2_. The 100 μl sample was added to the beads and incubated at 4°C for 1 h on a rotator before collecting the supernatant. The pellet was then washed with 2 × 0.5 ml HEPES pH 7.5, centrifuged and the supernatant was collected. Glycoproteins were eluted with 0.5 ml of 0.1 M L-fucose in PBS buffer, before a final elution with 50 μl 2X SDS loading buffer.

Jacalin (Jac) (Vectors Laboratories, Burlingame, CA, USA) (100 μl): The lectin beads were pre-equilibrated with 2 × 1 ml 175 mM Tris pH 7.5 before adding 100 μl of sample and incubating at 4°C for 1 h on a rotator, then collecting the supernatant by spinning down at 1,500 rpm. The pellet was washed with 2 × 1 ml 175 mM Tris pH 7.5, centrifuged at 2,000 rpm for 30 s and the wash buffer was collected. Glycoproteins were eluted using 1 ml 0.8 M galactose in 175 mM Tris pH 7.5 before a further elution with 50 μl 2X SDS loading buffer.

Peanut agglutinin (PNA) (Vectors laboratories, Burlingame, CA, USA) (100 μl): The lectin beads were pre-equilibrated with 2 × 1 ml 10 mM HEPES pH 7.5 and activated with wash buffer of 0.1 mM CaCl_2_ and 0.01 mM MnCl_2_ in 10 mM HEPES pH 7.5. A 100 μl sample was added to the beads and incubated at 4°C for 1 h on a rotator. The supernatant was then collected by centrifugation at 1,500 rpm. Lectin beads were washed with 1 × 1 ml 10 mM HEPES pH 7.5, centrifuged and the supernatant was collected. Bound glycoproteins were eluted with 1 ml 200 mM galactose in 10 mM HEPES pH 3.0 before a further elution with 50 μl 2× SDS loading buffer.

### Protein precipitation (TCA/DOC procedure)

The amylase depleted/glycoprotein enriched sample (1000 μl) was placed into a 1.5 ml centrifuge tube. Na-deoxycholate (2% DOC) (125 μg/ml) was added (0.85 μl) and vortexed at RT for 15 min. Then 33.3 μl 24% trichloroacetic acid (TCA) was added and vortexed for 2 min before centrifuging at 12,000 rpm for 10 min at 4°C. The supernatant was aspirated carefully then washed by centrifugation with 500 μl acetone (−20°C) to remove excess TCA, then the supernatant was discarded and the pellet resuspended in 50 μl 6 M urea, 50 mM Tris/HCl loading buffer. Pierce BCA protein assay (Thermo Scientific, Basingstoke, UK) was used for protein quantitation.

### SDS PAGE electrophoresis

Equal amounts of proteins were separated onto a criterion XT Bis-Tris gel 4–12% using XT MES running buffer (Biorad, UK). Samples were loaded according to protein quantitation and run at 25 mA/gel in 1× SDS running buffer. After the separation of the proteins, the gels were stained with Instant Blue (Expedeon Ltd, Harston, UK) for 10 min and transferred in distilled water for direct use or stored at 4°C overnight.

### In-gel digestion protocol

Gel bands were cut using a sterile scalpel then further cut into 1–2 mm^3^ gel pieces and transferred to a 1.5 ml tube. The gel was covered in 200 μl of destaining solution (50% methanol, 5% acetic acid in MilliQ-H_2_O) and shaken at RT overnight. The destaining solution was changed regularly until all the blue colour had been removed. The gel pieces were dehydrated using 200 μl acetonitrile for 5 min at RT and dried in a vacuum centrifuge for 2–3 min. Then 30 μl 10 mM dithiothreitol (DTT) buffer was added to reduce for 30 min at RT before adding 30 μl 50 mM iodoacetamide buffer to alkylate at RT for 30 min. Excess solution was removed before adding 200 μl acetonitrile to dehydrate the gel pieces for 5 min at RT until white coloration appeared. The excess acetonitrile was removed and the gel dried, then further dried in a vacuum centrifuge for 2–3 min (or until completely dry). The trypsin reagent was prepared by adding 1 ml of ice-cold 50 mM ammonium bicarbonate to 20 μg of trypsin (final concentration 20 ng/μl) and keeping the solution on ice. Then a 30 μl trypsin solution was added to the sample to rehydrate the gel pieces on ice for 10 min with gentle mixing. Gel pieces were spun down for 30 s and the excess trypsin solution was removed. Five μl 50 mM ammonium bicarbonate was added to the gel pieces and the digestion was carried out overnight at 37°C with gentle mixing. Fifty mM ammonium bicarbonate, 450 μl of extraction buffer 1 (50% acetonitrile, 5% formic acid in MilliQ-H_2_O) and 50 μl of extraction buffer 2 (85% acetonitrile, 5% formic acid in MilliQ-H_2_O) were successively added to the gel pieces for 10 min incubation and transferred into the same 1.5 ml tube. After the last incubation in extraction buffer 2, the sample was completely dried in a vacuum centrifuge, then resuspended in 20–40 μl of buffer A (98% MilliQ-H_2_O, 2% acetonitrile, 0.1% formic acid).

### Mass spectrometry

LC-MS/MS of peptides was performed using a Q-TOF 6520 (Agilent Technologies) equipped with a nanoLC-chip cube. The HPLC consisted of a nanoflow analytical and a capillary loading pump (Agilent 1200 series). Peptides were enriched and separated via nano-LC (0.075 × 150 mm, packed with Zorbax 300SB-C18, 5 μm material, 300 Å pore size) integrated in the HPLC Chip (G4240-62001). For each mass spectrometry experiment, peptides were loaded onto the enrichment column with 100% solvent A (2% acetonitrile with 0.1% formic acid). A two-step gradient generated at a flow rate 0.6 μl/min was used for peptide elution. This included a linear gradient from 5% to 40% buffer B (95% acetonitrile with 0.1% formic acid) over 45 min followed by a sharp increase to 100% B within 10 min. The total run time, including column reconditioning, was 60 min. Lockmass correction was performed using the poly (dimethylsiloxane ion ([M + H]1+ 445.1204 Da). Full scan was acquired over a range of m/z 400–1700 at 5 spectra per sec, and MS/MS over m/z 50–1700 at 2 spectra per sec selecting the 6 most abundant doubly or triply charged precursors per cycle. Spectra were deconvoluted and analysed using Spectrum Mill (Agilent). Auto-MS/MS was performed with a total cycle time of 3.3 s. Selected precursor masses were ignored for 0.9 min.

MS/MS spectra generated above were extracted from the raw data in mzXML file format using a converter from the Agilent trapper. The mzXML data were submitted to the in house version of the Trans-Proteomic Pipeline (TPP) [14] search for protein identification. Spectral Index Quantitation (SINQ) [15] was used for protein quantitation.

## Results

### Optimization of depletion strategies

In order to increase the number of less abundant proteins identified in saliva samples, a series of protocols to deplete highly abundant proteins from saliva were optimized.

#### Amylase depletion

**Figure 2:**
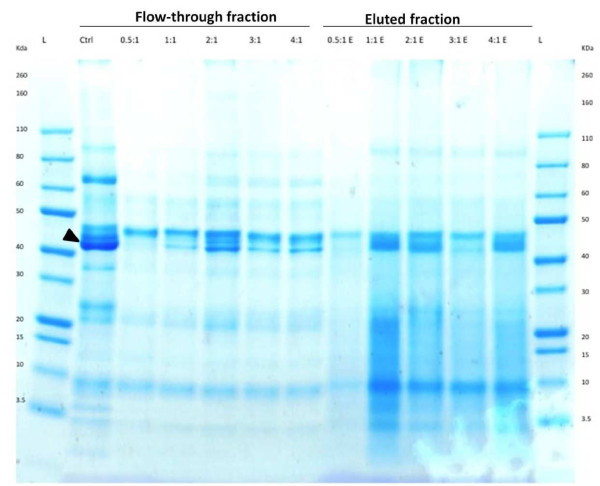
**Protein separation of saliva samples from a healthy control depleted of amylase using potato starch with different saliva (μL of saliva) to starch (μg of starch) ratios from 0.5:1 to 4:1.** The figure shows the flow through fractions and the eluted fractions using 2 × SDS loading buffer. Arrowhead denotes amylase band. Control denotes undepleted saliva sample from a healthy control. L = Ladder, E = Eluate, Ctrl = Control.

A starch-based depletion protocol was optimized and used to remove amylase from saliva. The resulting flow through fractions were then analysed by SDS-PAGE (Figure 2). A 1:1 (μl of saliva: mg of starch) was sufficient to significantly reduce the concentration of amylase. The efficiency of amylase depletion increased with higher saliva to starch ratios.

#### Lectin depletion

Different lectins, Concanavalin A (ConA), *Aleuria aurantia* (AAL) Jacalin (Jac), and Peanut Agglutinin (PNA) with various affinities for diverse sugar moieties were used to deplete abundant *N*-linked (ConA, AAL) and *O*-linked (Jac, PNA) glycoproteins from saliva. The previously mentioned lectins were used for depletion strategy optimization. The ratio of lectin (volume of lectin-conjugated beads) to the volume of amylase-depleted sample was optimized. The results are shown in Additional files 1, 2, 3 and 4). An optimal ratio of 1:2 (lectin to saliva, v: v) was found to efficiently enrich certain glycoproteins contained in saliva samples with minimal sample loss.

### Lectin efficiency

**Figure 3:**
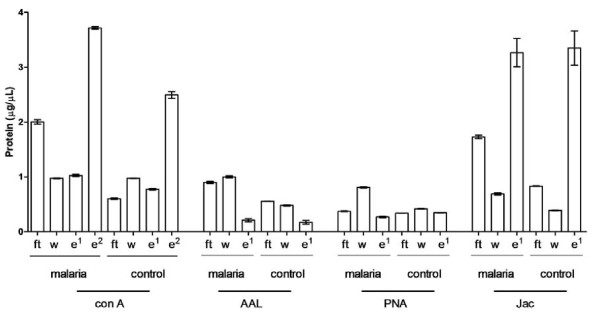
**Protein concentration obtained in each fraction of the Lectin depletion protocol in saliva samples.** The graph shows the total protein concentration (μg/μl) isolated in the flow-through fraction, wash and eluted fractions of lectin depletion protocols using ConA, AAL, PNA and Jacalin. Error bars indicate standard error of the mean. ft = Flow through, w = wash, e^1^ = First eluted fraction, e^2^ = Second eluted fraction (methyl α-glucoside).

The efficiency of the lectin depletion based on abundance was analysed by assessing the sample loss in the flow-through and elute fractions. The protein concentration of each fraction for the four lectins was measured using BCA assay protein quantitation. The ratio of total concentration of protein obtained in the eluted fraction to the total concentration of protein in the flow-through fraction was used to compare the lectin-binding efficiency. ConA and Jac showed the highest efficiency for overall glycoprotein enrichment, and *N*- and *O*-linked glycoproteins, respectively, as shown by the high concentration of protein recovered from the eluted fraction (Figure 3). AAL and PNA did not appear to be as effective at glycoprotein enrichment, which could indicate very tight binding requiring alternative elution conditions. An apparent optimal protocol, from an efficiency point of view, would combine the use of ConA and Jac. However, glycoprotein efficiency here is a measure of abundance and not the number of identifications displayed in Table 1 which suggests that AAL and not ConA is the better choice of lectin for *N*-linked glycoprotein depletion.

**Table 1:** Unique proteins identified in the flow through and eluted fractions of lectin depletion

Lectin	Sample	Flow-through fraction	Eluted fraction
**Concanavalin A**	**Control**	**74**	**25**
	Patient	123	30
**Jacalin**	**Control**	**82**	**22**
	Patient	219	16
**PNA**	**Control**	**71**	**13**
	Patient	85	18
**AAL**	**Control**	**108**	**18**
	Patient	171	25

### Mass Spectrometry analysis of depleted saliva samples

MS data was searched against a concatenated dataset of *Homo sapiens* and the 3D7 *P. falciparum* isolate protein sequences. A total of 1,955 peptides that corresponded to 192 proteins were identified at 1% false-discovery rate using a target-decoy strategy. Protein identifications of patients and control were compared using label-free methods.

Due to the low level of glycosylation of *P. falciparum* proteins, [16] the lectin flow-through fraction was expected to contain a higher number of parasite protein identifications. Eleven peptides that corresponded to four proteins assigned to *P. falciparum* were identified in the flow-through fraction of all lectins, namely PFL0480w, PF08_0054, PFI0875w and PF13_0119. However, due to sequence homology of these proteins with host proteins, only one parasite protein (PFL0480w) was identified in the saliva flow-through fraction with two or more peptides, and two parasite proteins (PF08_0054 and PFI0875w) were identified with one unique peptide, all of which could be unequivocally assigned to *P. falciparum* (not shared with host proteins).

The flow-through and eluted fractions were compared using four different lectins in patients and controls in order to identify candidate biomarkers found in the host proteome. Most proteins uniquely identified by LC-MS/MS in the saliva of malaria patients were plasma proteins.

CAP, adenylate cyclase-associated protein 1 (yeast)

Actinin, alpha 1

Stratifin

Coactosin-like 1

Calmodulin-like 5

Apolipoprotein A-I

Fibrinogen gamma chain

Superoxide dismutase 2, mitochondrial

Heat shock 27kda protein 1

Peroxiredoxin 2

S100 calcium binding protein A4

Carbonic anhydrase I

Haemoglobin, epsilon 1

Haemoglobin, zeta

Superoxide dismutase 1, soluble

Haemoglobin, gamma A

SPARC-like 1 (hevin)

Fibrinogen beta chain

Parkinson disease (autosomal recessive, early onset) 7

Ribonuclease T2

Vimentin

Enolase 1, (alpha)

Haemoglobin, delta

Haptoglobin-related protein

Haptoglobin

Carbonic anhydrase VI

Transketolase

Immunoglobulin heavy constant alpha 1

WD repeat domain 1

Lactotransferrin

Malate dehydrogenase 1, NAD (soluble)

Immunoglobulin heavy constant gamma 3 (G3m marker)

Comparison between malaria and non-malaria cases also identified a number of differentially regulated proteins (Tables 2 and 3). To minimize the false-discovery rate for proteins differentially regulated in saliva from malaria cases compared with non-malaria cases, data were compiled from each of the lectins and values were included only if proteins were up or down regulated for a particular protein with at least two different lectins.

**Table 2:** List of proteins identified both in malaria cases and in controls but increased in malaria patients

Proteins increased in malaria patients	fold increase
Peptidylprolyl isomerase A (cyclophilin A)	23.17
Aldolase A, fructose-bisphosphate	23.13
Azurocidin 1	22.96
Catalase	22.62
Haptoglobin	21.35
Calmodulin-like 3	21.19
Lymphocyte cytosolic protein 1 (L-plastin)	21.11
Prolyl 4-hydroxylase, beta polypeptide	20.98
Glucose-6-phosphate isomerase	20.95
Haemoglobin, beta	20.84
Hemopexin	20.64
Alpha-1-B glycoprotein	20.38
Histone cluster 2, h2be	20.25
Vesicle amine transport protein 1 homolog (T. Californica)	17.69
Heat shock 70kda protein 8	16.31
Transmembrane protease, serine 11D	15.97
Histone H4	15.94
Myeloperoxidase	15.92
Serpin peptidase inhibitor, clade B (ovalbumin), member 13	11.84
Ribosomal protein s27a	11.73
Actin, beta-like 2	11.58
Cystatin SN	11.36
S100 calcium binding protein A9	11.03
Capping protein (actin filament), gelsolin-like	8.87
Leucine-rich alpha-2-glycoprotein 1	8.82
Bactericidal/permeability-increasing protein-like 1	5.55
S100 calcium binding protein A11	5.24
Profilin 1	4.39
Lactoperoxidase	3.98
Alpha-2-macroglobulin	3.28
Rho GDP dissociation inhibitor (GDI) beta	3.07

**Table 3:** List of proteins identified in saliva that are decreased in malaria patients compared with non-malaria febrile controls

Proteins decreased in malaria patients	fold-decrease
Lipocalin 1 (tear prealbumin)	1.68
Thioredoxin	2.13
Single-chain Fv fragment	2.20
Dermcidin	3.03
Serpin peptidase inhibitor, clade B (ovalbumin),	3.22
Immunoglobulin heavy constant gamma 2 (G2m marker)	3.28
Immunoglobulin heavy constant mu	3.72
Immunoglobulin heavy constant alpha 1	4.65
Histone cluster 1, h1d	unique in controls

The analysis of the number and identity of the unique proteins obtained in the flow-through and eluted fractions were used to measure how effective and specific lectin-depletion was for patient and control saliva samples. The number of unique proteins identified in the flow-through and eluted fractions per lectin depletion strategy are shown in Table 1. AAL and Jac showed the most number of identifications in the flow-through fractions for *N*- and *O*-linked glycoproteins, respectively.

## Discussion

Lectin-depletion strategies in combination with amylase depletion are a simple and effective method to increase the number of proteins identified in saliva at low concentrations, and thus potentially useful for biomarker discovery studies in saliva. Although only two *P. falciparum* proteins were identified in this study, the proteomic signature of saliva from malaria positive cases revealed the up-regulation of a number of proteins associated with haemolysis and inflammation compared with febrile malaria-negative children.

This study provides further evidence that saliva is a non-invasive biological fluid suitable for the discovery of biomarkers of disease. The presence of highly abundant proteins in saliva, alpha-amylase in particular, is a clear obstacle to identify potential protein biomarkers that may be less abundant [3,17]. Thus, the optimization of depletion strategies to identify less abundant proteins is of paramount importance. Amylase-depletion was a quick and highly effective method to increase the number of proteins identified in saliva. The proteomic analyses of starch-depleted samples indicated the absence of amylase in the eluted and the flow-through fractions.

Due to the dynamic range of salivary proteins, additional strategies to enrich for less abundant proteins are essential. To accomplish this, the use of lectin-depletion methods was explored. The rational for the inclusion of a lectin-depletion step was to reduce at least partially the high proportion of glycoproteins found in saliva and to enrich for *P. falciparum* proteins. Although the extent of glycosylation of *Plasmodium* spp proteins is largely unresolved [16,18,19], there is evidence suggesting the absence of glycosylation (*N*- and *O*- linked) in *P. falciparum*. Indeed, the analysis of the *Plasmodium* genome demonstrates a lack of the necessary glycosyl transferases required to catalyse this reaction [16]. Removal of glycoprotein constituents of saliva using lectins was therefore included in the sample processing workflow to isolate potential, low abundant, non-glycosylated parasite proteins. We therefore expected non-glycosylated parasite proteins to be captured in the flow-through fraction of all four lectins using *N*- (ConA, AAL) and *O*-linked (Jac, PNA) specific lectins [11]. A number of peptides associated with malaria samples were shared by the host proteome and the protein sequences derived from the *P. falciparum* 3D7 isolate. However, only three proteins (PFL0480w, PF08_0054 and PFI0875w) could be unequivocally assigned to 3D7 proteins. Other peptide sequences for the proteins identified were shared by *P. falciparum* 3D7 isolate and *Homo sapiens*.

These results support the idea that lectin depletion is a useful method to increase dynamic range of protein identifications from saliva samples. Due to the apparent redundancy of the lectin-binding affinities, an optimal protocol may not necessarily include all four lectins. Indeed, Jac and ConA had the highest binding efficiency whereas PNA and AAL proved to be less efficient depletion strategies, suggesting that either the two lectins do not bind strongly or specifically to glycoproteins present in saliva. However, for this to be the case we would expect a larger concentration of protein to be captured in the flow-through fraction unbound which was not observed. Alternatively, it appears that non-specific or very strong binding may have occurred preventing the bound glycoproteins from subsequently being eluted by the competitive inhibitors. This is demonstrated in the second elution step in ConA which showed much higher protein concentrations using methyl α-glucoside after the first elution with methyl α-mannoside.

Identification of the proteins in the flow-through and eluted fractions of each lectin depletion protocol provided further evidence on the effectiveness of each strategy. Analysis of the number of unique proteins identified by each lectin showed that Jac and AAL identified the most number of unique proteins in the flow-through fractions of malaria patients for *O*- and *N*-linked glycoprotein depletion, respectively. For the latter, the majority of the reported *N*-glycans in saliva have been reported to be fucosylated on the core chitobiose structure contributing to the higher number of protein identifications in the flow-through of AAL than ConA [20]. Jac binds to T-antigen glycans (Galβ3GalNAc) with and without sialic acid residues whereas PNA is more selective towards non-sialylated structures [20]. The higher number of protein identifications in the flow-through fractions of Jac suggests a higher abundance of sialylated T-antigen glycans. Moreover, analysis of the protein identification of each lectin provided valuable guidance to optimize the depletion protocol. In addition to high abundance of α-amylase, Krief *et al.*[17] showed that other abundant proteins such as albumin and immunoglobulin may have similar “masking effects” on the identification of less abundant proteins. Immunoglobulin molecules are highly glycosylated and were effectively removed by lectins. However, 90% of albumin is not glycosylated [21], and mass spectrometry data showed its concentration was high in the flow-through of AAL, PNA, Con A and Jac, the eluted fraction of Con A and Jac but was undetectable in the eluted fractions of AAL and PNA. Adding a prior step to deplete albumin from saliva by sodium chloride/ethanol albumin depletion may prove a useful strategy to increase the identification of parasite proteins [22].

Analysis of the flow through fractions of patients and controls identified 31 proteins that were uniquely observed in the malaria patients. In particular, many of the host proteins identified are predominantly found in erythrocytes and 4 of 31 of the uniquely identified proteins were constituents of haemoglobin (haemoglobin epsilon 1, zeta, gamma A and delta). Peroxiredoxin 2 is the second most abundant erythrocyte protein after haemoglobin [23]. Other proteins that were unique or up-regulated in malaria cases like calmodulin, fibrinogen gamma chain and haptoglobin are associated with inflammation, endothelial vascular damage and haemolysis. Indeed, red-cell rupture and inflammatory response to parasite capture well the basic pathophysiology of malaria. However, we considered alternative explanations for these associations.

Fever is a clinical feature commonly accompanied by inflammation (increase vascular flow) of gums that could account for the leakage of plasma proteins into saliva. However, since saliva samples from non-malaria patients were also obtained from children with fever, it is unlikely that this mechanism accounts for the differences observed between patients and controls. It remains unclear whether plasma proteins found in saliva are a consequence of vascular damage induced by malaria parasites. Although the scope of this study was mainly to provide evidence to support the suitability of saliva to identify clinically useful biomarkers, the conclusion we can derive from our results are limited by a number of factors. Due to reduced sample volume available for these studies, we could not measure the absolute concentration of our candidate markers in individual samples or perform extensive fractionation for in-depth proteome analysis. This is a limitation of the study as the use of pooled samples does not exclude the influence of individual samples in the selection of candidate biomarkers. This limitation will be addressed in prospective studies.

Discovering biomarkers in saliva with a good diagnostic performance is clinically relevant. From an operational perspective, the rapid identification of malaria patients using saliva samples would immediately improve triaging practices in outpatient departments. Similarly, from a patient perspective we would anticipate that patient compliance would increase in clinical or surveillance studies that require frequent repeated sampling.

## Competing interest

All authors declare that they have no competing interest.

## Authors’ contributions

HH, MM, MC, EO, MLT carried out the proteomic studies and drafted the manuscript. BMK, DN and CCP participated in the design of the study, performed the statistical analysis. MM, EO, DN, EL and CCP conceived the study, and participated in its design and coordination. All authors read and approved the final manuscript.

## Supplementary Material

Additional file 1 **Lectin depletion using Concanavalin A (ConA) and optimization of starch depletion.** The figure shows the optimization of ConA depletion and starch depletion. (Key: L = Ladder, E = Elute, FT = Flow Through, W1 = Wash 1, W2 = Wash 2, AR = Amylase-removed Control, Ctrl = Control sample of saliva). Click here for file

Additional file 2 **Optimization of lectin depletion using AAL.** The figure shows the optimization of saliva sample volume, assessing the effectiveness of 50 μg lectin depletion of 50, 100, 200 and 400 μl of amylase removed saliva sample. The figure shows the differential band patterns in the FT and E showing successful depletion strategies. (Key: L = Ladder, E = Elute, FT = Flow Through, W = Wash, AR = Amylase Removed Control, AR-E = Elute from the Starch used to remove amylase, Ctrl = Control sample of saliva). Click here for file

Additional file 3 **Optimization of lectin depletion using jacalin.** The figure shows the optimization of saliva sample volume, assessing the effectiveness of 50 μg lectin depletion of 50, 100, 200 and 400 μl of amylase removed saliva sample. The figure shows the differential band patterns in the FT and E showing successful depletion strategies. (Key: L = Ladder, E = Elute, FT = Flow Through, W = Wash, SDS = Final Elute using SDS loading buffer, AR = Amylase Removed Control, Ctrl = Control sample of saliva). Click here for file

Additional file 4 **Optimization of lectin depletion using peanut agglutinin (PNA).** The figure shows the optimization of saliva sample volume, assessing the effectiveness of 50 μg lectin depletion of 50, 100, 200 and 400 μl of amylase removed saliva sample. The figure shows the differential band patterns in the FT and E showing successful depletion strategies. (Key: L = Ladder, E = Elute, FT = Flow Through, W = Wash, SDS = Final Elute using SDS loading buffer, AR = Amylase Removed Control, Ctrl = Control sample of saliva). Click here for file
