# Supplementary figures and images for: Proteomic identification of host and parasite biomarkers in saliva from patients with uncomplicated Plasmodium falciparum malaria
Source: Malar J. 2012 May 28;11:178. doi: 10.1186/1475-2875-11-178 (PMC3407698; doi:10.1186/1475-2875-11-178)

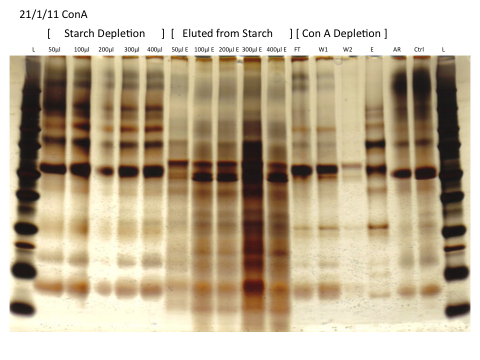

Supplement: Additional file 1 — Lectin depletion using Concanavalin A (ConA) and optimization of starch depletion. The figure shows the optimization of ConA depletion and starch depletion. (Key: L = Ladder, E = Elute, FT = Flow Through, W1 = Wash 1, W2 = Wash 2, AR = Amylase-removed Control, Ctrl = Control sample of saliva). [file 1475-2875-11-178-S1.docx]
