# Supplementary material for: Proteomic identification of host and parasite biomarkers in saliva from patients with uncomplicated Plasmodium falciparum malaria
Source: Malar J. 2012 May 28;11:178. doi: 10.1186/1475-2875-11-178 (PMC3407698; doi:10.1186/1475-2875-11-178)
Supplement: Supplementary file 2 [file 1475-2875-11-178-S2.docx]

L             FT 50μl        W 50μl      E 50μl        FT 100μl    W 100μl    E 100μl      FT 200μl   W 200μl     E 200μl     FT 400μl    W 400μl     E 400μl        AR              Ctrl           AR – E           L
