# Supplementary material for: Proteomic identification of host and parasite biomarkers in saliva from patients with uncomplicated Plasmodium falciparum malaria
Source: Malar J. 2012 May 28;11:178. doi: 10.1186/1475-2875-11-178 (PMC3407698; doi:10.1186/1475-2875-11-178)
Supplement: Supplementary file 4 [file 1475-2875-11-178-S4.docx]

FT 50μl     W 50μl       E  50μl      SDS 50μl    FT 100μl    W 100μl    E 100μl     SDS 100μl   FT 200μl   W 200μl     E 200μl   SDS 200μl   FT 400μl   W 400μl   E 400μl       SDS 400μl      AR         L
